# Rescue of follicle development after oocyte-induced ovary dysfunction and infertility in a model of POI

**DOI:** 10.3389/fcell.2023.1202411

**Published:** 2023-08-08

**Authors:** Sairah Sheikh, Belinda K. M. Lo, Heidy Kaune, Jassimran Bansal, Anna Deleva, Suzannah A. Williams

**Affiliations:** Nuffield Department of Women’s and Reproductive Health, Women’s Centre, John Radcliffe Hospital, University of Oxford, Oxford, United Kingdom

**Keywords:** oocyte, follicle development, primary follicle, granulosa cells, primary ovarian insufficiency, premature ovarian failure, infertility

## Abstract

The mechanisms and aetiology underlying the development of premature ovarian insufficiency (POI) are poorly understood. However, the oocyte clearly has a role as demonstrated by the Double Mutant (DM) mouse model where ovarian dysfunction (6 weeks) is followed by POI (3 months) due to oocyte-specific deletion of complex and hybrid N- and O-glycans. The ovaries of DM mice contain more primary follicles (3a stage) accompanied by fewer developing follicles, indicating a block in follicle development. To investigate this block, we first analysed early follicle development in postnatal (8-day), pre-pubertal (3-week) and post-pubertal (6-week and 3-month) DM (*C1galt1* ^F/F^ *Mgat1* ^F/F^:ZP3*Cre*) and Control (*C1galt1* ^F/F^ *Mgat1* ^F/F^) mice. Second, we investigated if transplantation of DM ovaries into a “normal” endocrine environment would restore follicle development. Third, we determined if replacing DM ovarian somatic cells would rescue development of DM oocytes. At 3-week, DM primary 3a follicles contain large oocytes accompanied by early development of a second GC layer and increased GC proliferation. At 6-week, DM primary 3a follicles contain abnormally large oocytes, accompanied with decreased GC proliferation. Transplantation of DM ovaries into a ‘normal’ endocrine environment did not restore normal follicle development. However, replacing somatic cells by generating reaggregated ovaries (ROs) did enable follicle development to progress and thus highlighted intra-ovarian factors were responsible for the onset of POI in DM females. Thus, these studies demonstrate oocyte-initiated altered communication between GCs and oocytes results in abnormal primary follicles which fail to progress and leads to POI.

## Introduction

Follicle activation and development are regulated by endocrine, paracrine, and autocrine signals (Adhikari and Liu, 2009; Grive and Freiman, 2015; Hsueh et al., 2015; Ford et al., 2020). More recently, biomechanics of the ovarian extracellular matrix (ECM) has been added to this group of crucial factors that regulate follicle development (Woodruff and Shea, 2007; Telfer et al., 2008; Ouni et al., 2020; Grosbois et al., 2023; Hopkins et al., 2021) It is understood that the more rigid cortex preserves primordial follicles (Chan et al., 2021) in a quiescent state (Nagamatsu et al., 2019; Biswas et al., 2022) whereas, the ‘softer’ medulla facilitates the growth and expansion of follicles (McLaughlin and McIver, 2009; Shah et al., 2018; Amargant et al., 2020; Kinnear et al., 2020). Thus, changes in the structure of the ovary have a direct effect on follicle behaviour (Xu et al., 2006; Woodruff and Shea, 2007; Kawamura et al., 2013; Kawamura et al., 2016; Lunde et al., 2001).

Ovarian dysfunction is also part of the etiology of premature ovarian insufficiency (POI, previously known as premature ovarian failure; POF). Two main etiological mechanisms, follicle depletion and follicle dysfunction, result in ovaries either being devoid of follicles (afollicular) or containing a spectrum of follicle development (follicular), respectively (Nelson et al., 1994; Meskhi and Seif, 2006; Nelson, 2009; Hubayter et al., 2010; Suzuki et al., 2015). Approximately 50% of women diagnosed with POI present with the follicular form (i.e., dysfunctional ovaries that still contain follicles) (Mehta et al., 1992; Nelson et al., 1994; Massin et al., 2004; Suzuki et al., 2015; Kaufman et al., 1981) which, due to the lack of inhibitory feedback by functional follicles, leads over time to the depletion of all follicles resulting in afollicular POI (i.e., ovaries without follicles). POI affects 1% of women under 40 years of age and is idiopathic in over 74% of cases (Coulam et al., 1986; Meskhi and Seif, 2006; Shelling, 2010), thus, the mechanisms that cause ovarian dysfunction are poorly understood (Shuster et al., 2010).

To investigate ovarian dysfunction and potential causes of POI, a mouse model exists that undergoes follicular POI as a result of oocyte-specific gene deletions and similarly to the human condition of POI, eventually afollicular POI. The model known as the Double Mutant (DM), exhibits a clearly defined timeline for the onset of POI. Females quickly transition from being subfertile at 6-week to infertile due to a lack of ovulation at 9-week and present with follicular POI by 3-month of age (Williams and Stanley, 2011). The DM model was determined as a model of POI based on parameters used to diagnose this condition in women; i.e., absence of developing follicles, ovary dysfunction, reduced testosterone and inhibin A and elevated FSH (Williams and Stanley, 2011). Follicular POI eventually leads to afollicular POI in DM mice (unpublished data) due to lack of follicular feedback as observed in women. The ovaries at both 9-weeks and 3-months contain decreasing numbers of developing follicles and increasing numbers of primary 3a follicles (no more than 20 granulosa cells), thus indicating a block in follicle development at this 3a stage since few follicles progress beyond it (Williams and Stanley, 2011; Grasa et al., 2012; Grasa et al., 2016). Follicle characterization revealed changes to the follicular basal lamina with increased levels of laminin and also reduced layers of theca cells (Grasa et al., 2016). Most recently, we have revealed that ovarian ECM gene expression alters rapidly with age (Kaune et al., 2022). The drop in fertility in DM females is accompanied by an altered endocrine profile (Williams and Stanley, 2011). This is rapid in comparison to women but enables us to investigate the etiology of POI.

This model sheds light on the role of the oocyte in ovarian function since the phenotype of the DM mouse results from oocyte-specific deletion of two glycosyltransferase genes, *C1galt1* and *Mgat1. C1galt1* encodes β1,3-galactosyltransferase (T-synthase) and *Mgat1* encodes *N*-acetylglucosaminyltransferase I (GlcNAcT-1). T-synthase is required for the generation of core 1-derived *O*-glycans (Ju et al., 2002a; Ju et al., 2002b), and GlcNAcT-1 for the synthesis of complex and hybrid *N*-glycans (Schlesinger et al., 1975; Robertson et al., 1978). The two alleles are floxed and deletion is initiated by the expression of a *Zp3Cre* transgene; ZP3 is expressed from the primary stage (Philpott et al., 1987). Therefore, the POI phenotype of DM females is due to oocytes lacking complex *O-* and *N-*glycans from the primary stage onwards. However, as there are two glycosylation defects, and many oocyte-generated glycoproteins contain either or both glycan, elucidating the molecules involved, and the specific intrafollicular effects that lead to follicle dysfunction and POI are ongoing.

Considering that both hormones and the ECM are altered in DM mice, we hypothesized these could contribute to the altered follicle development. Thus, the aim of this study was to perform morphometric spatiotemporal analysis of early follicles from postnatal (8-day), pre-pubertal (3-week), post-pubertal sub-fertile (6-week) and infertile POI (3-month) DM ovaries. Since the endocrine profile is also altered, we also assessed whether the ovarian dysfunction was the result of a non-supportive endocrine environment, since follicles with the genetic modification were originally capable of development. We then assessed whether the altered endocrine profile plays a role in the disarrayed follicle development by transplanting DM ovaries to a ‘normal’ endocrine environment. Finally, we assessed if DM germ and somatic cells’ communication is altered by replacing the DM ovary somatic cells using the reaggregated ovary (RO) technique.

## Materials and methods

### Mice

All experiments using mice were carried out with the approval of the Local Ethical Review Panel at the University of Oxford under license in accordance with the United Kingdom Animals (Scientific Procedures) Act 1986. Mice were provided with food and water *ad libitum* and maintained in individually ventilated cages in 12:12 h light-dark cycles.

Double mutant (DM) females carry floxed *C1galt1* and *Mgat1* alleles and a ZP3*Cre* transgene whereas females carrying the floxed *C1galt1* and *Mgat1* alleles but not the ZP3*Cre* transgene were used as Controls (the floxed alleles function as wildtype genes (Shi et al., 2004; Williams et al., 2007). For ovary transplant experiments, DM and Control females were selected as donors and Control siblings were used as recipients for the ovarian tissue.

For reaggregated ovary (RO) experiments, Control, DM or mice ubiquitously expressing the *LacZ* gene (Friedrich and Soriano, 1991) (generously provided by Prof. Elizabeth Robertson, University of Oxford) were used as the source of germ cells, and neonatal (P0-P1) B6SJLF1/J mice (WT) were used as a source of somatic cells. To produce B6SJLF1/J pups, C57BL/6 female mice (Harlan, United Kingdom) were mated with SJL/J males (Harlan, United Kingdom). Immunocompromised mice were used as recipients (C.B-17 SCID; Harlan, United Kingdom or B6Rag1; generously provided by Prof. Fiona Powrie, University of Oxford).

### Genotyping

DM and Control mice were genotyped using protocols as described (Grasa et al., 2012; Grasa et al., 2015).

### Ovarian histology and analysis

Ovaries and grafted tissue were collected and fixed in 10% buffered formalin (Sigma-Aldrich) for 6–8 h, before being paraffin embedded. ROs were fixed for 1 h in gluteraldehyde fixative (25% glutaraldehyde, 1 M MgCl_2_ in 0.1 M PBS), before washing 3 times for 15 min in 200 µL of detergent rinse (1M MgCl_2_, 1% sodium deoxycholate, 1% NP-40 in 0.1 M PBS. For staining, the tissue was incubated overnight in 100 µL of X-gal staining solution at 37°C followed by washing 3 times for 15 min in 200 µL of detergent rinse. Samples were then imaged, and then paraffin embedded. Embedded ovaries and grafted tissues were serially sectioned (5 µm) and every 10th section stained with hematoxylin (Shandon Gill 2 hematoxylin, Fisher Scientific, Loughborough, United Kingdom) and eosin (Sigma-Aldrich, Dorset United Kingdom) for analysis. In grafted ROs, all sections were used for follicle counts. Sections were imaged using a DM2500 Leica microscope (Microscope Services Ltd., Woodstock, United Kingdom) and a MicroPublisher 5.0 RTV camera (Qimaging, Microscope services Ltd.).

Only morphologically healthy follicles with a central oocyte and a visible nucleus were assessed and staged according to Grasa et al. (2015). Primary follicles were classified based on the number of GC in a cross section as either primary follicles if they had a single cell layer (3a follicles; 9–20 GCs, 3b follicles; 21–60 GCs) according to Pedersen and Peters (Pedersen and Peters, 1968), or early secondary follicles (e2°) if they had two complete layers but less than 61 GC. Follicle area (excluding theca cells) and oocyte area (excluding zona pellucida) were measured using ImageJ (National Institutes of Health, Bethesda, Maryland, United States) with the follicle basement membrane was used as the follicle boundary. For an accurate linear regression analysis 3b and e2° follicles with 21–55 GCs were analysed; based on the maximum GC number for all ages. GC area was obtained by subtracting oocyte area from follicle area. To calculate the packing density, GC area was divided by the number of GCs.

### Immunohistochemistry

Immunohistochemistry (IHC) was used to detect the presence of Ki67. Briefly, sections were dewaxed with xylene and rehydrated using decreasing concentrations of ethanol and ddH_2_O. Slides were washed in Tris Buffered Saline (TBS: 0.1 M Tris pH 7.5 and 0.3 M NaCl) with 0.05% Tween-20 (TBST). Endogenous peroxidase was quenched by washing with 3% H_2_O_2_ (Fisher Scientific, Loughborough, United Kingdom) in Phosphate Buffered Saline (PBS) for 5 min. Sections were blocked with 1.5% normal goat serum (Vectastain ABC Kit, Vector Labs, Peterborough, United Kingdom) in TBS for 1 h and then incubated with rabbit anti-Ki-67 antibody (ab66155; Abcam, Cambridge, United Kingdom) at 1:100 overnight at 4°C; control sections were incubated with normal goat serum 1.5%. After 3 washes with TBST, sections were incubated with biotinylated anti-rabbit IgG secondary antibody (Vectastain ABC Elite Kit, Vector Laboratories) for 1 h, followed by ABC solution (Vectastain ABC Elite Kit, Vector Laboratories) for 30 min. A 3,3′-Diaminobenzidine (DAB) peroxidase substrate kit (Vectastain ABC Elite Kit, Vector Laboratories) was used to develop the stain. The slides were counterstained with haematoxylin, dehydrated, mounted with DEPEX (VWR, Leicestershire, United Kingdom) and imaged. For analysis of Ki67 staining, GCs were classified as either Ki67 negative or positive using an assessment scale (see results).

### Ovary transplantation under the kidney capsule

Ovaries for transplantation were collected from mice killed by cervical dislocation and placed in warm sterile L-15 Leibovitz media (Hyclone, Thermo Scientific, Utah, United States) supplemented with 3 mg/mL of BSA. The bursa and any additional non-ovarian tissue were carefully removed. DM and Control ovaries were fragmented and matched according to size before being surgically transplanted under the kidney capsule. The transplanted ovarian tissue was recovered 4 weeks after the transplantation, and processed as described above.

### Generation of reaggregated ovaries (ROs)

The generation of a RO involved the dissociation of ovaries into germ and somatic cells followed by the exchange and combination of both cellular types to form a reaggregated pellet; known as an RO. The procedure was carried out as described previously (Lo et al., 2019) with some modifications. Ovaries from seven to 10 adult lacZ females (6–8 weeks), seven to ten adult DM and Control females (9-weeks), or four to six neonates (P0-2) were collected into warm PBS supplemented with 1 mg/mL BSA (BSA fraction V, Thermo Fisher Scientific, Loughborough, United Kingdom). Ovaries were enzymatically dissociated into a single cell suspension with 0.05% trypsin and 0.53 mM EDTA (GIBCO), supplemented with 0.02% DNase-I (Sigma-Aldrich, Dorset United Kingdom) for 15 min at 37°C and 5% CO_2_ in air. The single cell suspension was transferred into a 15 mL centrifuge tube with an equal volume of warm Medium 199 (M199, GIBCO) supplemented with 31.3 mM sodium lactate (Sigma), 10 mg/mL Penicillin-Streptomycin (P0781, Sigma) and 10% fetal bovine serum (FBS; Biosera), centrifuged at 663 *g* for 5 min (Baird et al., 2004; Donnez et al., 2006). The supernatant was discarded, and the pelleted cells were resuspended in fresh M199/FBS transferred to a 35 × 10 mm cell culture treated dish for overnight culture. The following morning the non-adhered cell population was separated from the adhered cells; non-adhered cells included germ cells, non-viable somatic cells and red blood cells. The germ cell population was filtered using a 30 µm pre-separation filter (Miltenyi Biotec GmbH, Germany) in order to exclude the more developed oocytes and this was transferred to a new tissue culture dish with fresh M199/FBS. The remaining somatic cell monolayer was washed three times with PBS/BSA to remove any residual unattached cells. The adhered somatic cells were then treated with trypsin for 5 min at 37°C, collected, centrifuged at 663 *g* for 5 min and resuspended in fresh media. Cells were transferred into a new cell culture-treated dish and cultured for further 6 h. The second round of differential adhesion and separation was carried out as before to ensure complete separation of cell types. Germ and somatic cells were collected, centrifuged and re-suspended in 100 μL of M199/FBS and cells counted using a haemocytometer.

To generate RO, newborn somatic cells were combined with adult germ cells; phytohemagglutinin (PHA-P; Sigma) was added to create a final concentration of 35 μg/mL to promote cell cohesion. The suspension was centrifuged at 10,000 × *g* for 30 s, the tube was then rotated 180° and centrifuged for a further 30 s. The cell pellet is now referred to as a RO. ROs were transferred with onto a polycarbonate Transwell membrane (6.5 mm diameter, 0.4 µm pore; Corning) in a 24-well tissue culture treated plate (Corning Costar) for overnight culture in Waymouth MB 752/1 media (Sigma) supplemented with 10% FBS. Following overnight culture, ROs were surgically transplanted beneath the kidney capsule of a bilaterally ovariectomised immunocompromised mouse for 21 days; follicle development in mice takes 21 days.

Recipients were anesthetised with isoflurane and the surgical area prepared by removing the fur with clippers and cleaning with chlorhexidine solution. The anesthetised animal was placed on a heated pad at 37°C and injected subcutaneously with 0.05–0.1 mg/kg of buprenorphine (Vetergesic, Alstoe Limited, York, United Kingdom) to provide analgesia after surgery. Under magnification, the left kidney was exteriorised through a dorso-lateral incision and a small opening on the kidney capsule made using a pair of fine tip forceps. The pellet was placed beneath the kidney capsule and the kidney was returned to the body cavity. Next, a bilateral ovariectomy was carried out. Using fine tip forceps, the bursa was opened and the ovary exposed. The ovarian vasculature was ligated using 5.0 silk suture (Vicryl, Ethicon, US) and the ovary removed. The body wall was closed with interrupted suture using absorbable suture material. Using the same skin incision, the opposite side of the body wall was opened and the contralateral ovariectomy performed, followed by the suturing of the body wall and skin with interrupted suture (Vicryl, Ethicon, US).

Mice were placed in a warm recovery chamber until fully recovered. During the first 48 h after surgery, mice had easy access to food pellets, mashed pellets, water and medicated jelly with meloxicam (Metacam, Boehringer Ingelheim, Bracknell, UK) to provide analgesia. At 21 days after transplantation, mice were killed by cervical dislocation, and the kidney exposed and the RO collected into PBS on ice.

### Statistical analysis

Statistical analyses were performed using Prism GraphPad software (GraphPad Software, Inc., version 4.0 b, 2004. La Jolla, CA, United States). D’Agostino and Pearson normality test was applied to test Gaussian distribution of samples size n > 10. Where sample size n < 10, the Shapero-Wilk test of normality was used. The unpaired *t*-test was used to analyse differences between samples with normal distribution whilst the Mann–Whitney *U* test was performed to detect differences between groups with non-parametrical distribution and equal variances and a Welch’s correction was applied to groups with non-parametrical distribution and unequal variances. To assess correlation between GC number and area, if normally distributed, the Pearson correlation coefficient was tested; for non-parametric distribution, the non-parametric Spearman correlation was tested. Differences between GC number and area at the different ages were analysed using linear regression. To compare GC packing density and mean area between ages, a one-way ANOVA with Tukeys’ multiple comparison was used. A few groups were not normally distributed, for these the Kruskal Wallis with Dunn’s multiple comparisons test was used. Unpaired *t*-test was used to assess differences in theca cell area whilst a Kruskal Wallis with Dunn’s multiple comparison test was used to compare theca cell numbers. A one-way ANOVA followed by Dunn’s multiple comparisons test was used to analyse proportion of Control and DM follicles in whole ovary *versus* ROs and to analyse the mean number of follicles per RO. Results are presented as mean ± SEM. *p* ≤ 0.05 was considered significant.

## Results

### 3a follicles: Analysis of GC packing density

**FIGURE 1:**
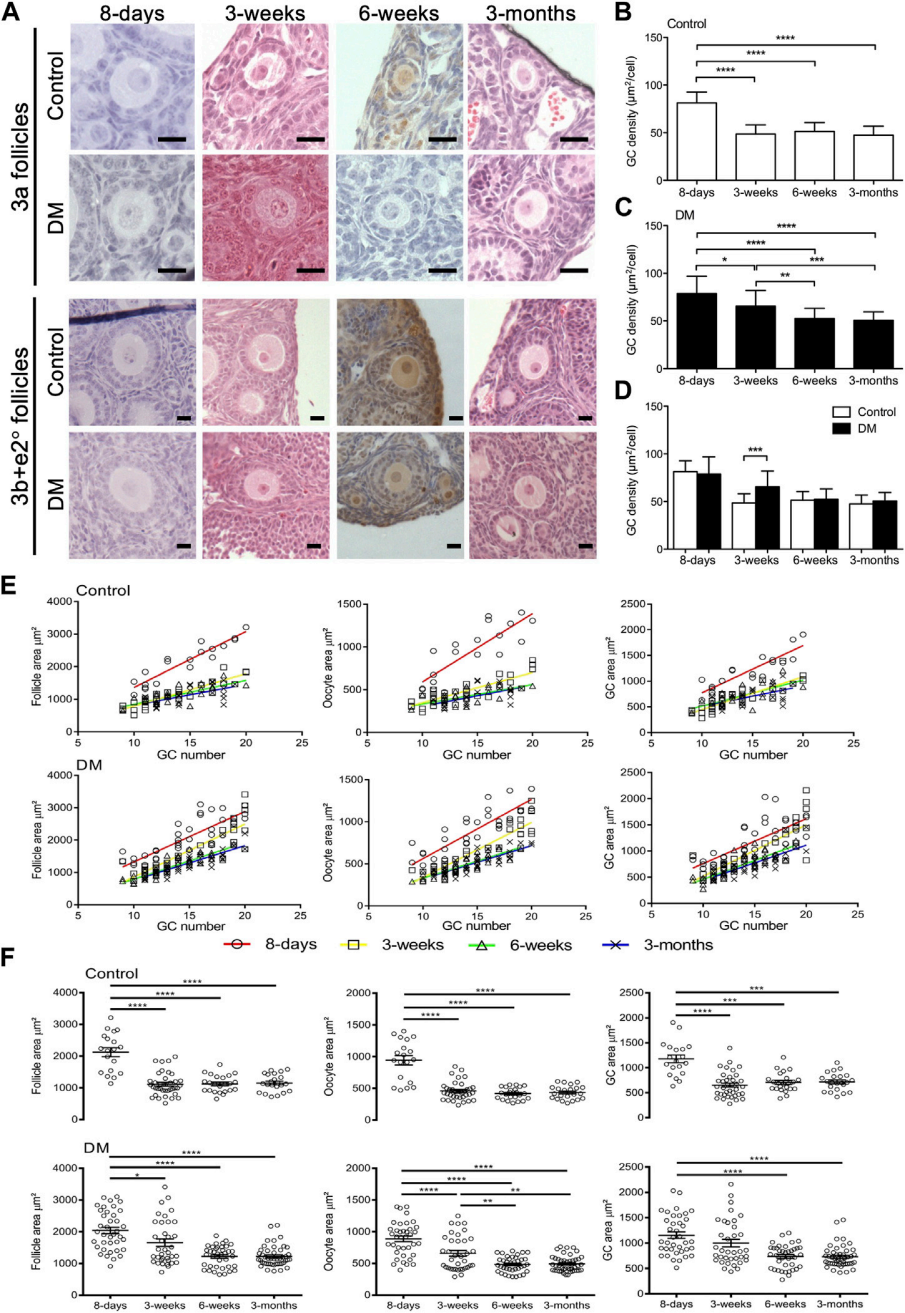
Analysis of early follicles in Control and DM ovaries. **(A)** Representative images of primary follicles (3a, and 3b and early secondary; e2°) in Control and DM ovary sections at 8-day, 3-week, 6-week, and 3-month. Scale bar = 20 μm. Granulosa cell (GC) packing density of **(B)** Control and **(C)** DM follicles at 8-day, 3-week, 6-week and 3-month. **(D)** Comparison of GC packing density between Control and DM at the different ages. **(E)** The relationship between follicle, oocyte and GC area with GC number in 3a primary follicles in Control and DM at 8-day (open circles; Control *n* = 3 mice, *n* = 19 follicles, DM *n* = 3 mice, *n* = 38 follicles), 3-week (open squares; Control *n* = 3 mice, *n* = 37 follicles, DM *n* = 3 mice, *n* = 36 follicles), 6-week (open triangles; Control *n* = 3 mice, *n* = 23 follicles, DM *n* = 3 mice, *n* = 38 follicles) and 3-month (crosses; Control *n* = 3 mice, *n* = 22 follicles, DM *n* = 3 mice, *n* = 46 follicles). **(F)** Comparison of follicle, oocyte and GC area of primary 3a follicles within Control and within DM ovaries at the different ages. Results are expressed as mean ± SD. *p ≤ 0.05, **p ≤ 0.01, ***p ≤ 0.001, ****p ≤ 0.0001.

As this study focused on determining how the oocyte affected early follicle development, we analysed all follicles less than 60 GCs; 3a (1 layer with 9–20 GCs), 3b and e2° (1 to 2 layers with 21–60 GCs) with a visible nucleus at 8-day, 3-week, 6-week and 3-month in Control and DM ovaries. Follicles from 8-day ovaries appeared larger than follicles from older ovaries (Figure 1A). To investigate these morphological differences, GC density was calculated and compared between the different ages. In Control ovaries (Figure 1B) GC density was significantly larger in 8-day ovaries compared to the other older ages. However, in DM ovaries, the reduction in GC density was less abrupt with gradual but significant declines from 8-day to 3-week to 6-week before levelling off (Figure 1C). Comparison between the Control and DM ovaries revealed that the sole difference between the genotypes was a change in GC density in follicles at 3-week (Figure 1D).

### 3a follicles: Analysis of follicle, oocyte and GC area with age

Next, we investigated the relationship between the follicle, oocyte, and GC area to GC number in 3a follicles. In both Control and DM ovaries at all ages, follicle, oocyte, and GC area increased proportionally with GC number (Figure 1E). Although area increased as expected with GC number, 3a primary follicles from 8-day ovaries (red) appeared to be larger as first observed from images and followed a different growth trajectory to follicles from the other ages. Linear regression revealed that there were significant differences in slopes for Control between all areas (follicle, oocyte and GC) at 8-day and all other ages, whilst no differences in the slopes existed between the other ages (Supplementary Table S1). Whereas, for DM, a significant difference in the slopes existed when comparing follicle and oocyte areas at 8-day (red) to 6-week (green) or 3-month (blue). However, when comparing GC area and all areas (follicle, oocyte, and GC) at 8-day (red) to 3-week (yellow) although the slopes did not differ, the intercepts did. Further differences in areas existed between 3-week to 6-week and 3-month (Supplementary Table S1).

Delving further, we found that Control 3a primary follicles at 8-day were larger, with an increased oocyte and GC area than those from older ages (Figure 1F). No further differences were observed when comparing 3a primary follicles from the older ovaries (3-week, 6-week and 3-month). In DM 3a follicles, a similar pattern to Controls was observed with a decline in oocyte size, follicle size and GC area at 8-day compared to older ovaries (Figure 1F). However, similar to the GC density, the decline in these three variables was less abrupt in DM ovaries of increasing age with 3-week follicles showing an intermediate phenotype.

### 3a follicles: Comparison of areas between Control and DM ovaries

Direct comparison between Controls and DM 3a follicles was carried out ascertain at what age the lack of oocyte glycans was modifying follicle development. No differences in follicle, oocyte and GC area between Control and DM were observed at the earliest (8-day) or the latest (3-month) age analysed (Supplementary Figure S1). However, at 3-week of age, DM follicles were larger than Control follicles with an increase in oocyte and GC areas (Supplementary Figure S1), this difference in oocyte size was also observed in 3a follicles at 6-week of age (Supplementary Figure S1).

### 3b + *e*2° follicles: Analysis of follicle, oocyte or GC area

Having examined the developmental characteristics of 3a follicles, where the block in development occurs in DM mice, we analysed development of follicle containing 20–60 GCs, to ascertain if the changes observed in 3a follicles persisted. For 3b + *e*2° follicles, when comparing Control and DM for the three variables (follicle, oocyte and GC area), interestingly, no differences were found at any of the 4 ages (Supplementary Figure S1).

We also investigated if there were differences in follicle development with age. Similar to Control 3a follicles, Control 3b + *e*2° follicles from 8-day ovaries were larger with an increased follicle and GC area than the older ages. The oocyte also was larger in Control 3b + *e*2° follicles at 8-day compared to 3-week and 6-week but surprisingly, not at 3-month where the variability in the size of the oocyte increased dramatically with a trend towards an increase in size (Supplementary Figure S1). In DM 3b + *e*2° follicles, a similar pattern to 3a follicles was observed with age (Supplementary Figure S1). Although there was an overall reduction in size of the three variables with age, the decline was much less pronounced than observed in Controls due to a more modest change at 3-week.

As expected, were exploring these variables in line with GC number of 3b + *e*2° follicles, all three increased with GC number in Control and DM at all ages (Supplementary Figure S1).

### Analysis of GC layers and cell proliferation

**FIGURE 2:**
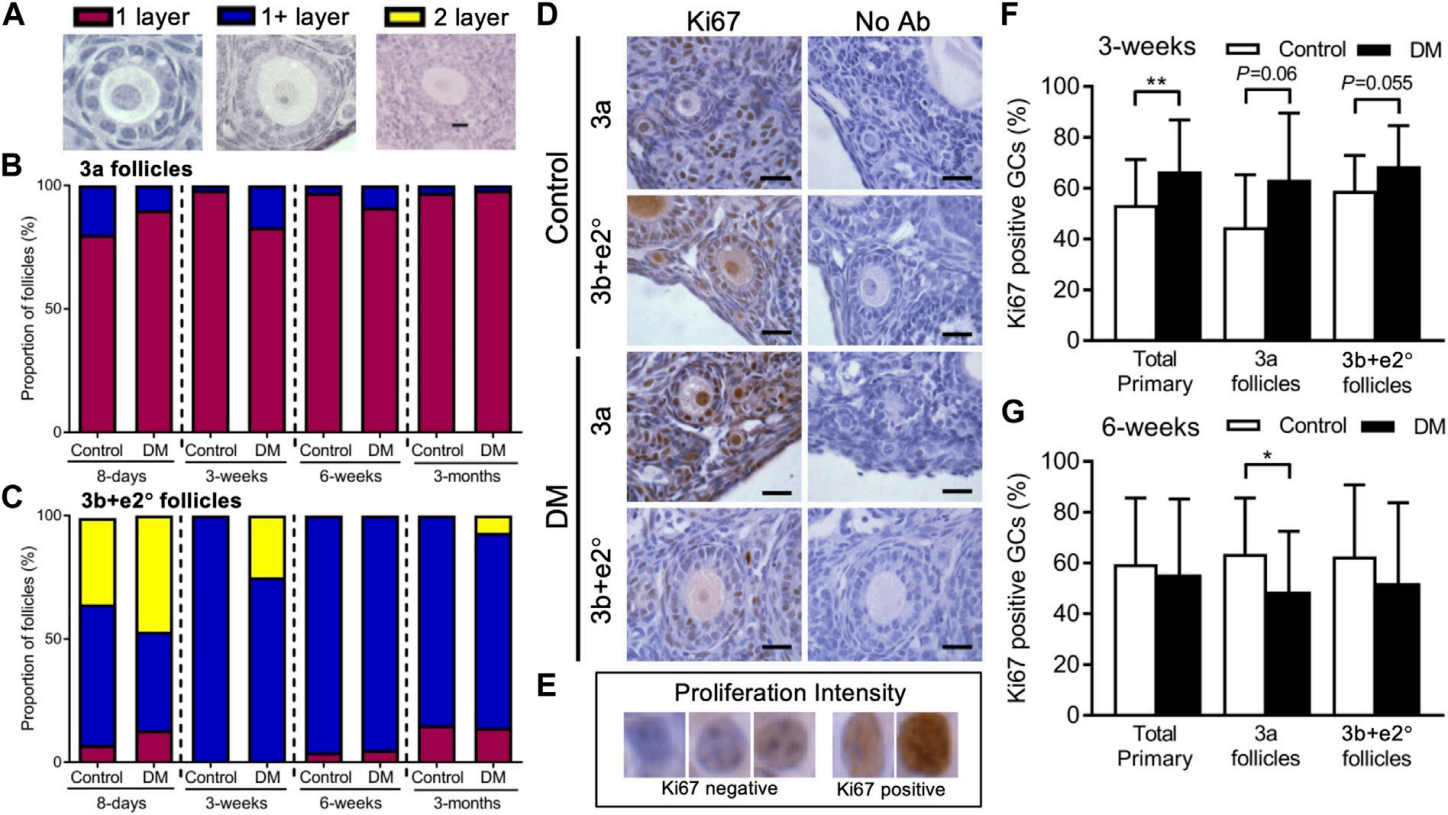
Analysis of early follicle granulosa cell layer formation and cell proliferation in Control and DM ovaries. **(A)** All early follicles (less than 61 granulosa cells: GC) were classified as follicles with 1 layer of GC (1 layer), follicles that had started developing the second layer (1+ layers) or follicles that had developed 2 complete layers (2 layers). The proportion of **(B)** 3a (20 GC or less) and **(C)** 3b and early secondary (e2°) follicles (21–60 GC) classified with 1 layer (magenta), 1+ layer (blue) and 2 layers (yellow) were analysed. **(D)** Representative images with the proliferation marker Ki67 labelling in GCs of 3a and 3b + *e*2° follicles from Control and DM ovaries. **(E)** GC proliferation intensity was used to assess percentage of Ki67 positive GC cells in follicles from **(F)** 3-week and **(G)** 6-week Control (3-week total follicles *n* = 28, 3a follicles *n* = 11, 3b + *e*2° follicles *n* = 17; 6-week total follicles *n* = 33, 3a follicles *n* = 16, 3b + *e*2° follicles *n* = 14) and DM ovaries (3-week total follicles *n* = 34, 3a follicles *n* = 13, 3b + *e*2° follicles *n* = 21; 6-week total follicles *n* = 50, 3a follicles *n* = 28, 3b + *e*2° follicles *n* = 14). Scale bar = 25 μm *p ≤ 0.05, **p ≤ 0.01.

**FIGURE 3:**
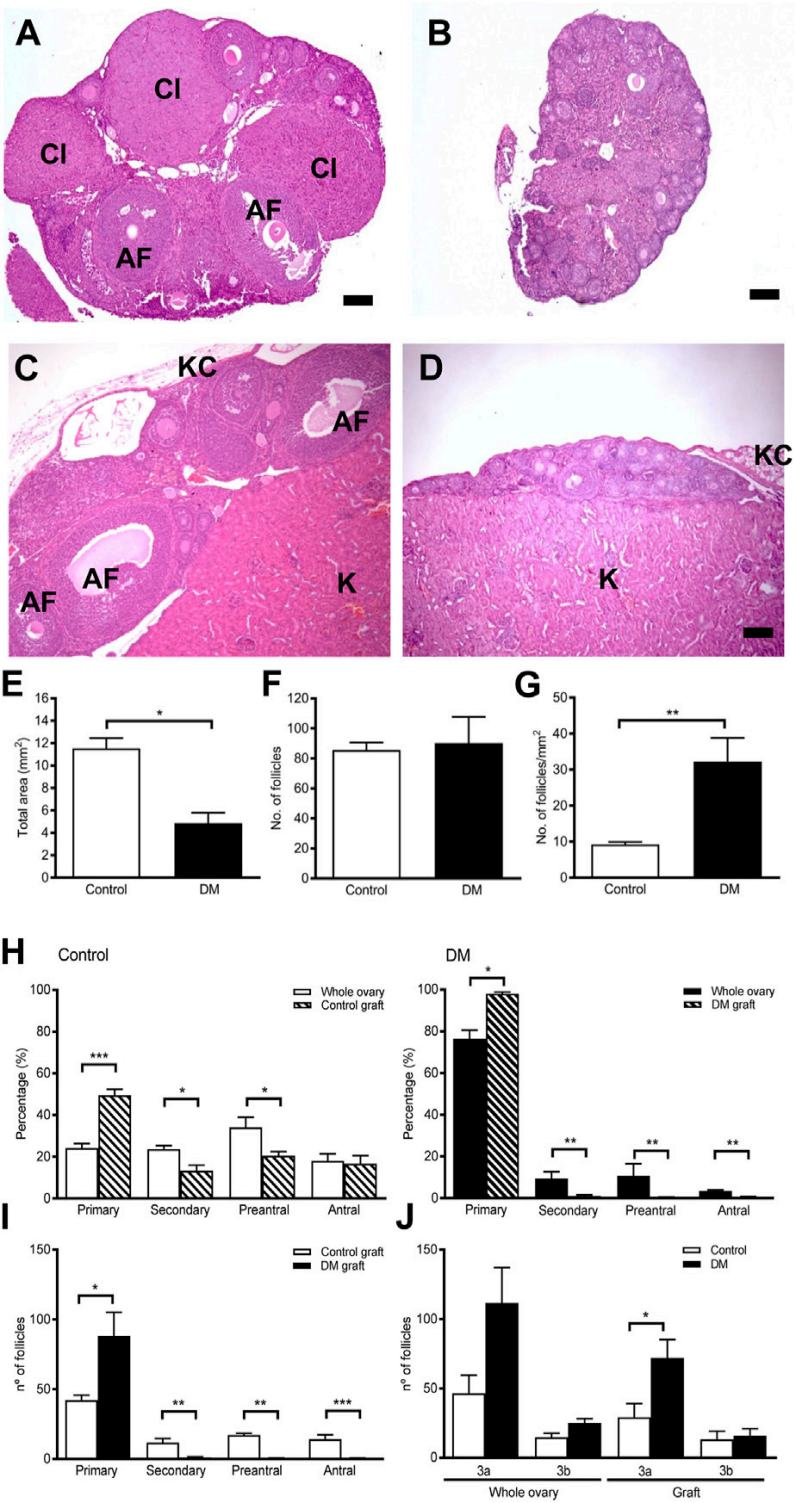
Ovarian morphology and follicle development in ovarian tissue graft from Control and DM mice. **(A)** Representative image of a Control 9-week ovary containing numerous follicles at different stages of development alongside corpora lutea. **(B)** Representative image of a DM 9-week ovary containing only primary follicles. **(C)** After 4 weeks of ovarian tissue transplantation to a wildtype immunocompatible host, Control grafted ovarian tissue had normal ovarian morphology containing all stages of follicle development whilst. **(D)** DM grafted ovarian tissue exhibited morphology comparable to a 9-week DM ovary. AF: antral follicle, Cl: corpus luteum, **(K)** kidney tissue, KC: kidney capsule. Bar scale represents 100 um. **(E)** Total area in the sections analysed of grafted ovarian tissue (Control *n* = 5 and DM *n* = 8). **(F)** Number of follicles counted in each ovarian graft by analysis of every 10th section. **(G)** Density of follicles per mm^2^ of grafted ovarian tissue analysed in Control and DM. **(H)** Proportion of follicles at each stage of development in Control and DM 9-week ovaries (Control *n* = 3, DM *n* = 3) and in graft tissue (Control *n* = 5, DM *n* = 8). **(I)** Comparison of the number of follicles at each stage of development in the Control and DM ovarian tissue graft. **(J)** Number of primary follicles Figure 3A, B: Control and DM whole ovary and ovarian tissue graft. Results are expressed as mean ± SEM. *p < 0.05, **p < 0.01, ***p < 0.001.

Due to the differences in follicle development, we investigated cell proliferation and the development of granulosa cell layers in follicles. To determine if follicles at the different ages were developing faster (i.e., were they developing a second layer sooner), follicles were classified as either follicles with 1 layer of GCs (1 layer), follicles that had started developing the second layer (1+ layers) or follicles that had developed 2 complete layers (2 layers) (Figure 2A). The proportion of 3a follicles (Figure 2B) and 3b + *e*2° follicles (Figure 2C) in these categories was analysed.

We first confirmed that a higher proportion of 3a and 3b + *e*2° follicles in Controls had 1+ and 2 layers respectively from 8-day ovaries than at older ages. At 3-week of age, a higher proportion of DM 3a (Figure 2B) and 3b + *e*2° follicles (Figure 2C) had 1+ layer compared to Controls, suggesting DM follicles were developing differently, which is consistent with the larger follicles observed in DM 3-week ovaries (Figure 1D).

Since differences in area between Control and DM follicles existed at 3- and 6-week, and follicles from DM 3-week ovaries develop a second layer of GC earlier, we examined whether these changes were accompanied by changes in GC proliferation (Figure 2D). The proportion of Ki67 positive GCs per follicle were analysed in all primary follicles and for 3a and 3b + *e*2° follicles separately using the GC assessment scale (Figure 2E).

At 3-week of age (Figure 2F) when DM ovaries contain larger follicles than Control, the proportion of Ki67 positive GCs was higher in all primary follicles from DM ovaries than Controls however when separated into 3a and 3b + *e*2° follicles, significance was not quite achieved when comparing Control to DM.

Since in 6-week DM ovaries we do not see an increase in follicle or GC area accompanying the presence of large oocytes (Figure 1F **),** we examined changes in GC proliferation (Figure 2G). Although no differences were observed in the percentage of Ki67 positive GCs in all primary follicles, when split into 3a and 3b + *e*2°, DM 3a follicles contained a lower proportion of proliferating GCs compared to Controls; no differences existed at the 3b + *e*2° stage. Therefore, although the oocyte continues to grow this is not accompanied by an increased proportion of proliferating GC surrounding DM oocytes in 3a follicles, and thus as observed, overall follicle size does not vary between Control and DM (Supplementary Figure S1).

### A normal endocrine environment is unable to restore follicle development in DM ovarian tissue

Control ovaries from mice at 9 weeks of age contain follicles at all stages of development as well as multiple corpora lutea whereas DM ovaries contain only small follicles (Figures 3A,B). To determine if a normal endocrine environment could facilitate the resumption of DM ovarian follicle development, ovarian tissue from 9-week DM or Control mice was transplanted under the kidney capsule of immunocompatible sibling Control females for 4 weeks.

Analysis of the Control grafted tissue (Figure 3C) revealed morphology comparable with 9-week Control ovaries (Figure 3A), with the presence of many follicles including large antral follicles. Similarly, the morphology of DM grafted tissues (Figure 3D) was equivalent to 9-week DM ovaries (Figure 3B) with even fewer follicles beyond the early stages of development than the whole ovary.

Although ovarian tissue from both Control and DM mice were matched according to size when transplanted, the Control grafted tissues were significantly larger than DM when retrieved 4 weeks after transplantation (Figure 3E). However, total follicle counts were comparable in both groups and consequently the follicular density of the grafted tissue, represented as total number of follicles per mm^2^, was significantly higher in DM than Controls due to the smaller size of the tissue (Figures 3F,G).

In Control grafts, all follicle stages were present comparable to Control ovaries confirming that the host endocrine environment supported ovarian function and follicle growth (Figure 3H). On the contrary, in DM grafts follicles did not develop (Figure 3H), and thus DM grafts contained fewer developing follicles than Control grafts (Figure 3I). Since DM ovaries contain more 3a follicles due to a block in development (Williams and Stanley, 2011; Grasa et al., 2016) (Figure 3J), we investigated the number of primary follicles at the 3a and 3b stages in the grafts. The grafts mirrored this distribution pattern with more 3a follicles in DM grafts compared to Control grafts whilst 3b follicle numbers did not differ. Therefore, follicle development in the DM grafted ovaries is not normalised by exposure to a normal hormonal environment as the block at the 3a stage of follicle development still exists in the grafted ovaries.

Since normalising the endocrine environment did not rescue DM follicle development, and yet the genetic deletion occurs in the oocytes at the same stage, we hypothesised that the function of the surrounding somatic cells and/or ovarian environment had been altered and thus we tested if replacing them with naïve wildtype cells using the reaggregated ovary (RO) technique would restore follicle development.

### Formation of ROs using adult germ cells

**FIGURE 4:**
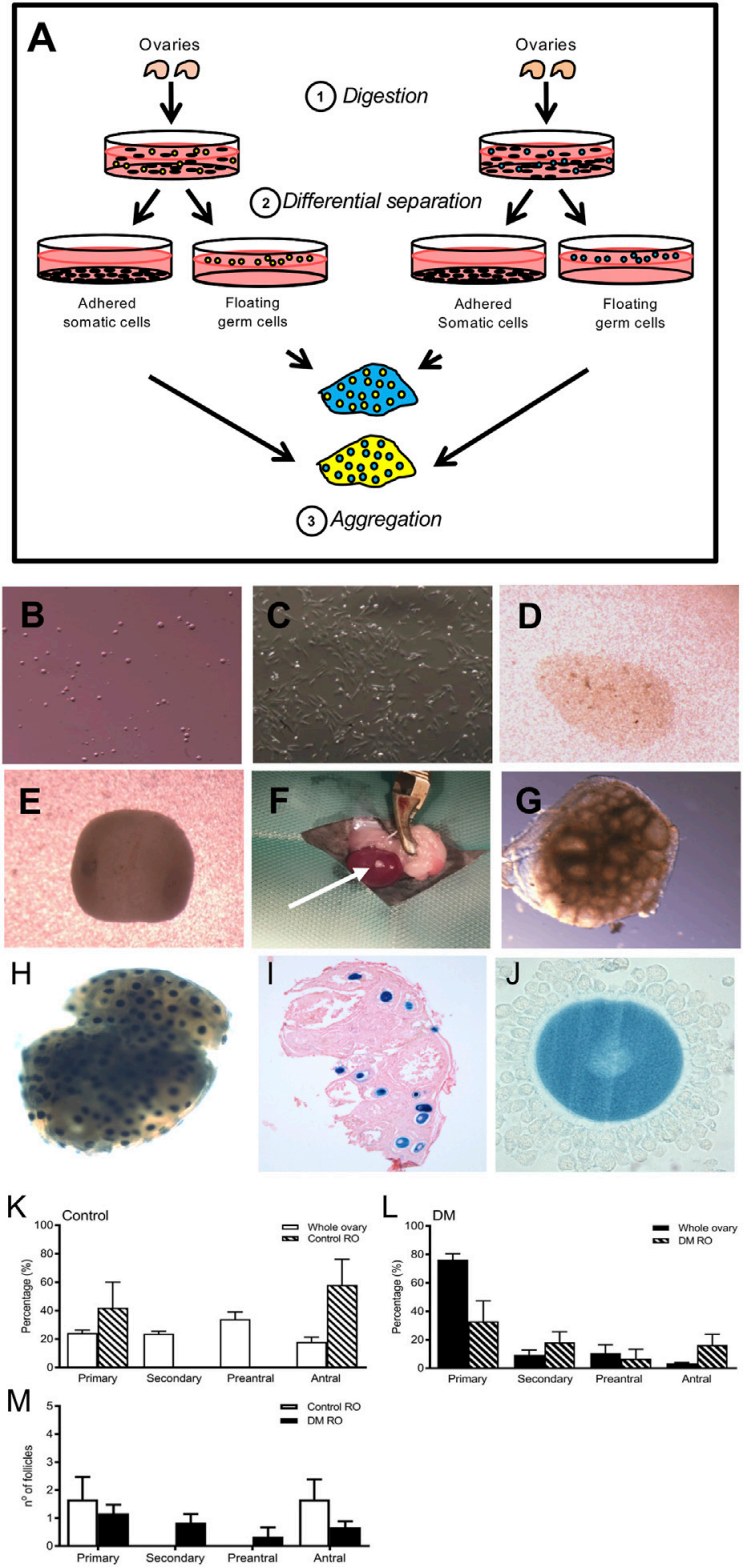
Replacement of somatic cells by using a reaggregated ovary to assess DM germ cell function **(A:1)**. Ovaries are digested into a single cell suspension **(A:2)**. During incubation, the somatic cells and germ cell populations are differentially separated since somatic cells adhere whereas the germ cells do not. **(A:3)** Isolated somatic cell populations and germ cells from two different sources are aggregated to form a pellet which after overnight culture is transplanted into an immuno-compromised mouse to support follicle growth and development. Image of isolated **(B)** germ and **(C)** somatic cells following differential separation. The 2 cell types are combined in the desired ratio and centrifuged to form a **(D)** reaggregated pellet which is left to culture overnight where it rounds up **(E)**. ROs are transplanted under the kidney capsule for 21 days **(F)**: (arrow points to the RO after 21 days of transplantation). **(G)** Upon retrieval, ROs contain follicles at various developmental stages as observed by the ‘bubbles’ in the tissue. **(H)** To demonstrate clean separation of somatic and germ cells, ROs were generated using germ cells from mice ubiquitously expressing *LacZ* and wild type somatic cells. After incubation with X-gal, germ cells from *LacZ* mice are stained blue and somatic cells are unstained. **(I)** Representative image of histological section confirming 100% of oocytes were from the *LacZ* mice (counterstained with eosin; *n* = 5). **(J)** High magnification image of a cumulus-oocyte-complex showing blue oocyte and unstained cumulus cells. Comparison of the proportion of follicles at each stage of development in **(K)** Control 9-week whole ovaries (*n* = 3) and Control ROs (*n* = 5) and (L) DM 9-week whole ovaries (DM *n* = 3) and DM ROs (DM *n* = 6) (M) Comparison of follicle numbers between Control and DM ROs. Results are expressed as mean ± SEM.

Briefly, the generation of a RO (Figure 4) first involves the isolation of ovarian somatic and germ cells from different sources, followed by their exchange and combination to form a reaggregated pellet (Figures 4A1–3). This pellet is transplanted under the kidney capsule of an ovariectomised immunocompromised mouse for 21 days, which supports follicle development (Figures 4A–G).

To confirm efficient separation of adult germ cells and newborn somatic cells, ROs were generated using ovaries from 6–8 weeks old mice ubiquitously expressing *LacZ* gene and neonatal wildtype (WT) mice. Labelling cells expressing *LacZ* using X-gal revealed blue staining of the oocytes and histological analysis revealed no blue somatic cells thus all somatic cells originated from WT ovaries, demonstrating clean somatic cell and germ cell isolation using adult LacZ ovaries (Figures 4H–J).

DM germ cells are able to generate follicles in a reaggregated ovary and overcome primary follicle arrest.

ROs were generated using germ cells from 9-week old Control or DM ovaries and newborn wildtype somatic cells (referred to as either Control or DM ROs, respectively). Both Control and DM ROs were transplanted into immunocompromised mice for development. Upon retrieval, they were fixed, sectioned and all follicles counted and staged.

Control ROs contained developing follicles and these were distributed between the primary and antral stage (Figure 4K). Remarkably, ROs generated using germ cells from infertile 9-week DM ovaries contained follicles at later stages of development as opposed to DM ovaries that contained predominantly primary follicles (Figure 4L). In fact, DM ROs were able to generate follicles to the same extent as Control ROs (DM: 4.5 ± 1.1 and Control: 3.3 ± 0.9, DM *n* = 6 ROs, *n* = 27 follicles, Control *n* = 6 ROs, *n* = 20 follicles).

Comparison of follicle numbers between Control germ and DM germ ROs revealed no differences (Figure 4M), however the number of follicles that developed in both DM and Control ROs were low compared to whole ovaries. Despite this, since DM ROs contained follicles at all stages of follicle development, this indicated that the block at primary development was overcome by replacement of the somatic cells.

## Discussion

Whilst follicle dysregulation is one of the known causes of POI (Knauff et al., 2009), the mechanisms and aetiology underlying the development of POI are unknown. In the DM mouse model of POI, DM females show an age-dependent decrease in fertility; being sub-fertile at 6-week of age producing one small litter, infertile by 9-week of age due to a lack of ovulation and undergo POI by 3-month of age; initially the ovary contains follicles and is thus follicular POI but degenerates to afollicular POI over time. Follicle development is also modified with an increase in primary follicle numbers accompanied by a depletion of developing follicles, indicative of a block in follicle development (Williams and Stanley, 2011; Grasa et al., 2012; Grasa et al., 2016). Here we showed that DM ovaries contain developing follicles with abnormally large oocytes, concomitant with decreased GC proliferation suggesting altered communication between oocytes and GCs. Our data also highlights transplantation of DM ovaries into a ‘normal’ endocrine environment was unable to rescue the altered follicle development indicating the abnormal follicle development is likely due to intrinsic ovarian factors. Finally, these results are corroborated by the analysis of DM germ cell function using the RO highlighting indeed intra-ovarian factors were responsible for the onset of POI in DM females. Overall, our results indicate altered communication between GCs and oocytes is responsible for the follicular distribution observed within the DM ovary.

This study revealed interesting differences in normal early follicle development and how this is affected by age. Primary follicles from 8-day ovaries are larger in size, with larger oocytes than primary follicles from 3-week, 6-week and 3-month Control ovaries. Several studies have revealed there are two distinct populations of primordial follicles, arising from Foxl2-or Lgr5-positive somatic cells, and these play different roles in ovarian development and reproductive function (Mork et al., 2012; Zheng et al., 2014; Faire et al., 2015; Fu et al., 2018; Niu and Spradling, 2020; Soygur and Laird, 2021). Foxl2-positive primordial follicles are activated during the first wave immediately after birth and are responsible for the establishment of the hypothalamic-pituitary-gonadal axis; likely those observed in 8-day ovaries, whilst Lgr5-positive primordial follicles are activated during the second wave and establish the lifetime ovarian reserve. The differences in follicular developmental dynamics observed may also be attributed to differences in GC origin and in location of follicle growth. Follicles from 8-day ovaries, which arise from ovarian surface epithelium and develop in the medulla, grow faster than follicles from the older ovaries, which are located in the cortex (Pedersen, 1970; Lunenfeld et al., 1975; Hirshfield, 1991; Byskov et al., 1997; Da Silva-Buttkus et al., 2008; Zheng et al., 2014; Buffler and Roser, 1974; Byskov, 1974; Nakano et al., 1977; Peluso and Steger, 1978; Fukuda et al., 1997) These differences in sources of GCs and location of growth, may be affecting the developmental dynamics of follicle growth with age as observed in our study (Parrott and Skinner, 1999; Eppig, 2001; Durlinger et al., 2002; Donnez et al., 2004; Gilchrist et al., 2004; Gilchrist et al., 2008; Dolmans et al., 2010; Peng et al., 2013; Donnez et al., 2015).

Interestingly, these follicular developmental patterns were also modified in DM ovaries. Since the genetic defect is oocyte specific, the oocyte must be having an effect on the development of the surrounding granulosa cells. Thus, oocyte glycans are affecting the granulosa cells in the first wave of follicle development more than the second. This has implications for how follicle development differs between pre- and post-pubertally. Moreover, the discovery of how the absence of glycans affects follicle development at different ages, may have implications for the future development of follicle culture when using tissue from patients of different ages.

The altered endocrine profile observed in postpubertal DM females is consistent with POI (Williams and Stanley, 2011) and could potentially be an important factor contributing to the quiescent state of the DM ovaries and lack of follicle development past the 3a stage. In this sense, a normal hormonal environment could restore follicle development and ultimately fertility in this mouse model. Therefore, we assessed the effect of a normal hormonal environment on DM ovarian follicle growth *in vivo*.

In Controls, all stages of follicle development were observed 4 weeks after transplantation confirming that the environment provided by the host supported ovarian function and follicle growth. In stark contrast, analysis of DM grafted ovaries revealed markedly hypotrophy of the tissue compared to Control grafts with a lack of follicle development. Therefore, although follicles survived in DM grafted tissue, DM follicles are unable to develop. These data indicate that the mechanisms underlying the onset of POI in the DM females is complex and is not restricted to the abnormal endocrine environment observed in these females. The fact that the deletion of *Mgat1* and *C1galt1* genes is restricted to the oocyte, points towards abnormal communication between the oocyte and the surrounded granulosa cells as the triggering event that impacts deeply on ovarian physiology.

It is clear that the physiological conditions in which follicles grow play a vital role in regulating their development; e.g., alginate follicle culture systems demonstrate that more rigid environments restrict follicle growth (Xu et al., 2006; West et al., 2007). Upregulation in components of the ECM stroma such as collagen, laminin and HA have been linked to increased ovarian stiffness and age-related fibrotic changes (Grasa et al., 2016; Shah et al., 2018; Amargant et al., 2020; Ouni et al., 2020; Mendez et al., 2022). Previously, we have shown DM follicles have a modified BL accompanied by increased laminin content (Grasa et al., 2016) and an upregulation of ECM genes (Kaune et al., 2022) and thus there are clear changes in the DM ovarian ECM. A rigid ECM impedes ovulation and CL regression; which involves remodeling of the ovarian ECM (Amargant et al., 2020; Mara et al., 2020; Ouni et al., 2020). In line with this, in DM we have previously observed the presence of luteinised unruptured as a result of failed ovulation following hyperstimulation and a failure in CL regression (Grasa et al., 2016). Further signs of altered DM somatic and germ cell communications are apparent in this study. We reveal discrepancies from as early as 3-week since DM ovaries at this age contain abnormally large 3a follicles and oocytes, accompanied by accelerated development of a second GC layer and increased GC proliferation. Since the genetic modification is oocyte-specific and occurs at the same point in follicle development—primary, there must be a change in the surrounding environment for follicle development to differ with age. Therefore, we assessed if replacing the DM ovarian somatic cells with naïve wildtype cells would restore follicle development using the RO technique (Lo et al., 2019). Generation and analysis of ROs enables us to determine the separate influence of the somatic and germ cells on follicle development and was established using newborn somatic and germ cells from mice up to 12 days of age (Eppig et al., 2000; Eppig and Wigglesworth, 2000; Eppig et al., 2002). Here, we have implemented this technique for the first time using germ cells isolated from adult ovaries.

ROs produced using 9-week Control or DM germ cells were able to generate follicles at all stages of development, from primary to antral. This was an unexpected finding since at 9-week of age DM females are infertile (Grasa et al., 2012) with a lack of follicles developing to the later stages. These findings confirm our hypothesis, that modifying the oocyte environment using the RO technique rescued the ability of DM germ cells to coordinate follicle development. The fact that DM 9-week oocytes retain the ability to sustain follicle development, when associated with WT somatic cells indicates that the genetically altered oocyte is inducing changes in the physiology of the somatic cell compartment resulting in aberrant follicle development and oocyte growth.

**FIGURE 5:**
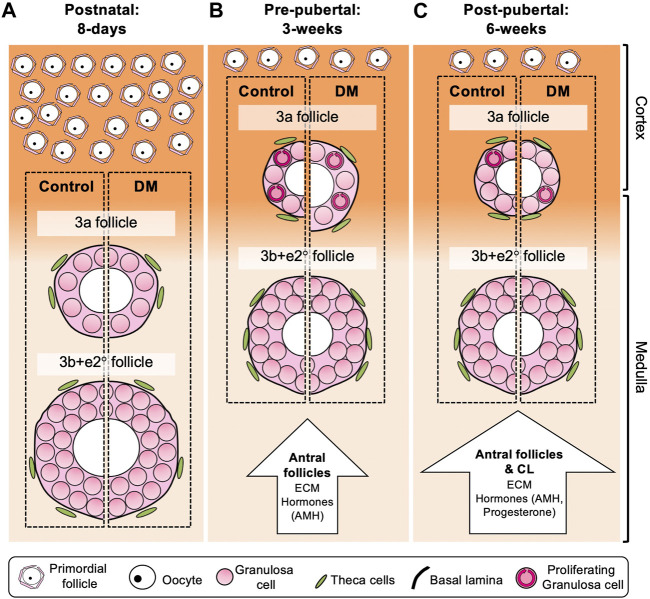
Model of follicle development in postnatal (8 days), pre-pubertal (3 weeks) and post-pubertal (6 weeks) Control and DM ovaries. Comparison of Control follicles between the different ages revealed that 3a and 3b + *e*2° follicles in the postnatal **(A)** ovary were larger than follicles from pre- and post-pubertal **(B, C)** ovaries, whilst no differences were observed when comparing these follicles in 3 weeks and post-pubertal ovaries. In postnatal ovaries **(A)**, early follicles develop throughout the ovary including the medulla (pale orange), which is less rigid and thus potentially more permissive to follicle growth than the cortex (dark orange) resulting in large 3a and 3b + *e*2° follicles in both Control and DM ovaries. In contrast, 3a follicles in pre-pubertal **(B)** and post-pubertal **(C)** ovaries are smaller than those in the postnatal ovaries potentially due to differences in the ovarian environment. These 3a follicles develop predominantly in the cortex and cortex:medulla interface which is a more rigid environment. The presence of antral follicles in pre-pubertal ovaries **(B)**, and both antral follicles and corpora lutea in post-pubertal ovaries **(C)** lead not only to upregulation of ECM molecules such as laminin and collagen around the follicles, but are also a source of hormones such as AMH and progesterone which affect follicle growth. When comparing DM to Control, although follicle growth was equivalent in postnatal ovaries **(A)**, in pre-pubertal ovaries **(B)** DM 3a follicles had increased oocyte, follicle and GC area, which was accompanied by increased GC proliferation (circular arrows within GCs). In post-pubertal ovaries **(C)**, DM primary 3a follicles had increased oocyte area only compared to Control 3a follicles, accompanied by less GC proliferation. No differences in 3b + *e*2° follicles were observed between Control and DM at any age. Therefore, this model depicts the specific changes that occur in DM 3a follicles as mice age reflecting 3a follicle dysfunction and the initiation of POI, whereas if the follicles manage to develop to 3b + *e*2°, subsequent follicle development is normal.

We propose a model based on the data described in this paper to demonstrate how early follicle development changes with age, and between Control and DM (Figure 5). In this study, we found developing follicles from both Control and DM postnatal (8-day) ovaries (Figure 5A) were larger than follicles in pre-pubertal (Figure 5B) and post-pubertal ovaries (Figure 5C). These differences can be attributed to differences in location of follicle growth, since follicles are located in the medulla of the postnatal ovary rather than the cortex as observed in the pre- and post-pubertal ovaries. In addition, differences exist in the composition of the postnatal ovary with the absence of large antral follicles and CL which may be restricting follicle growth in the older ovaries, both physically, due to the upregulation of ECM, and through the secretion of hormones; incorporating previously published data. When comparing Control to DM, whilst no differences exist in postnatal ovaries (Figure 5A), differences exist in follicle dynamics of 3a follicles in the pre-pubertal (Figure 5B) and post-pubertal ovaries (Figure 5C). Precocious follicle growth is observed in DM 3-week ovaries (Figure 5B) with 3a follicles being larger in size oocyte, follicle and GC area than Controls accompanied by increased GC proliferation. Finally, post-pubertal DM ovaries (Figure 5C) contain abnormally large oocytes in 3a follicles, which are not accompanied by increases in GC area due to decreased GC proliferation. No differences are observed in 3b + *e*2° follicles.

In conclusion, these studies demonstrate that oocytes from infertile DM females with POI retain the ability to develop into mature follicles when associated with WT somatic cells. Our results highlight hitherto unknown complex interactions between the oocyte and the somatic cells by various glycans, and reveal the potential for ROs as a clinical therapeutic tool to recover follicle development in women with POI and enable them to have their own genetic children.

## Data Availability

The raw data supporting the conclusion of this article will be made available by the authors, without undue reservation.
