# Supplementary figures and images for: Rescue of follicle development after oocyte-induced ovary dysfunction and infertility in a model of POI
Source: Front Cell Dev Biol. 2023 Aug 8;11:1202411. doi: 10.3389/fcell.2023.1202411 (PMC10443433; doi:10.3389/fcell.2023.1202411)

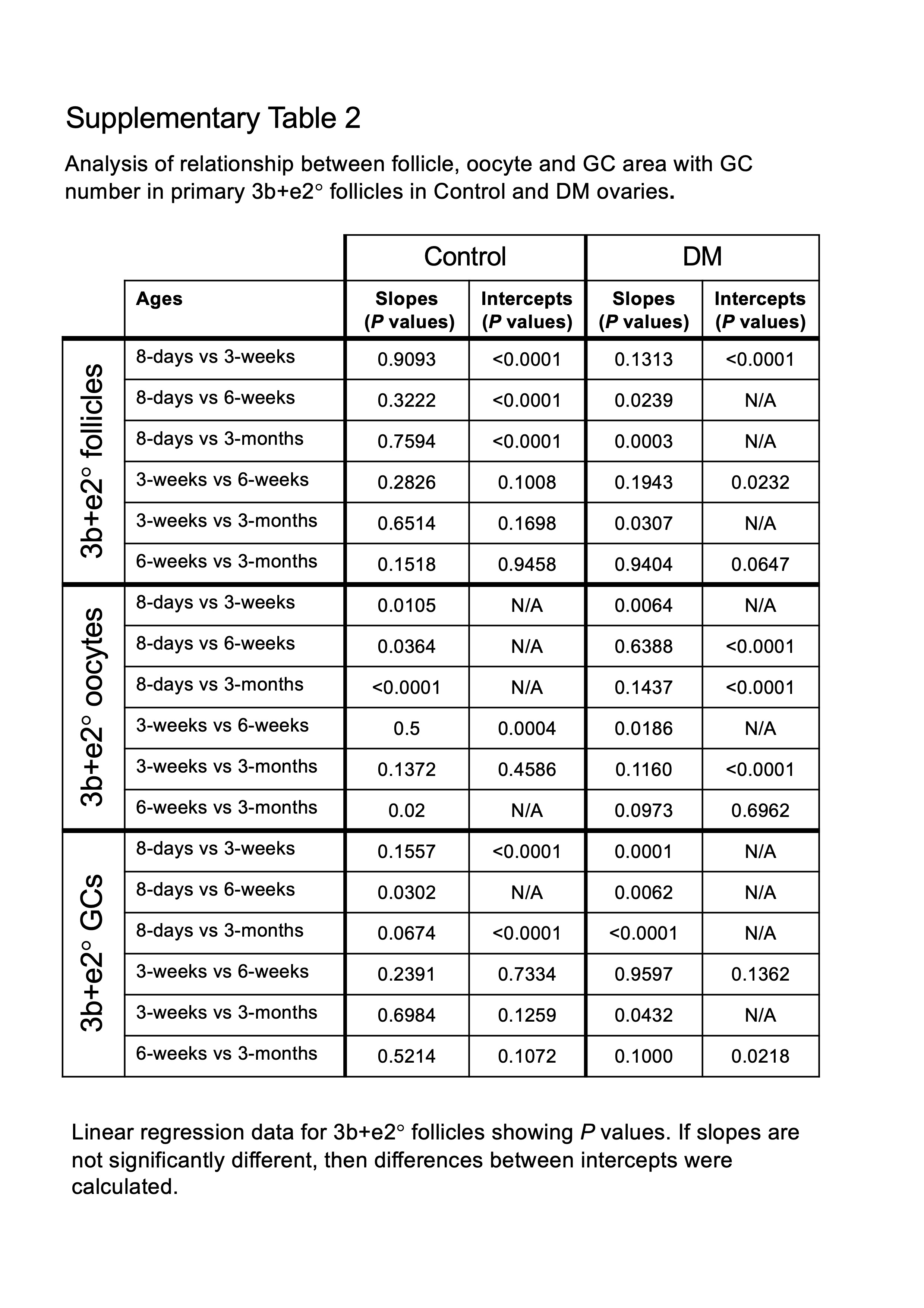

Supplement: Supplementary file 1 [file Image3.JPEG]

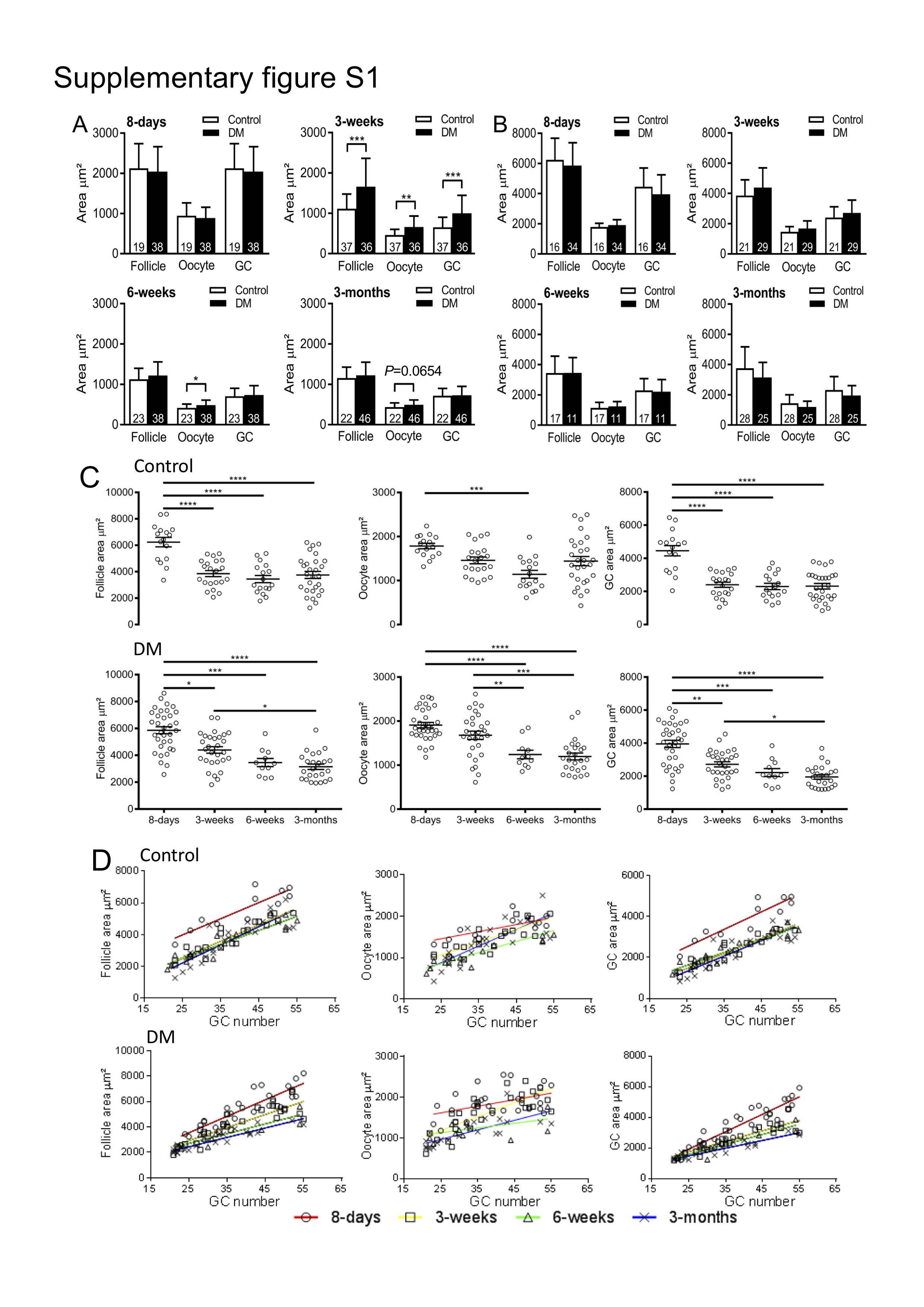

Supplement: Supplementary file 2 [file Image1.TIFF]

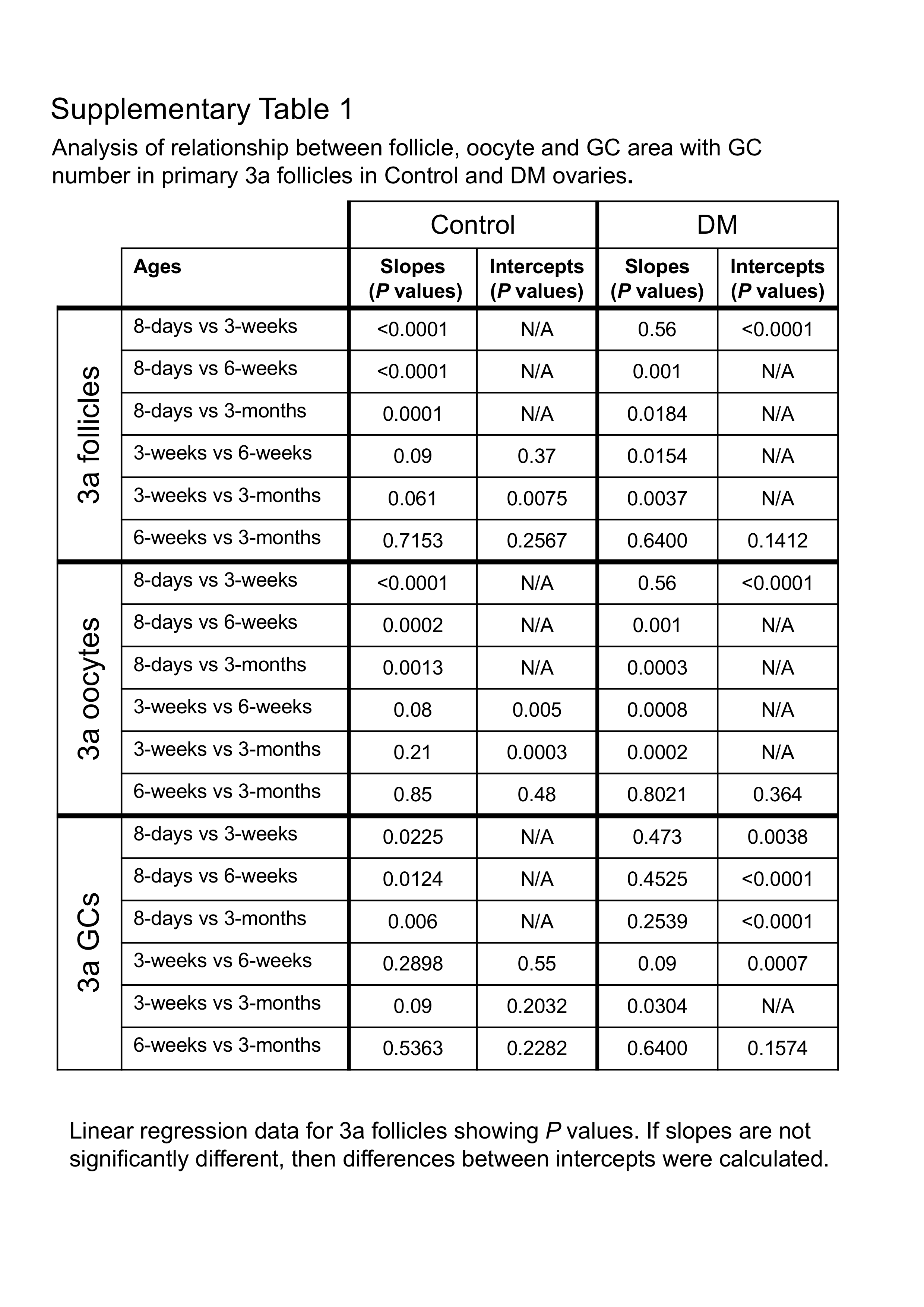

Supplement: Supplementary file 4 [file Image2.TIFF]
