# Supplementary material for: Rescue of follicle development after oocyte-induced ovary dysfunction and infertility in a model of POI
Source: Front Cell Dev Biol. 2023 Aug 8;11:1202411. doi: 10.3389/fcell.2023.1202411 (PMC10443433; doi:10.3389/fcell.2023.1202411)
Supplement: Supplementary file 3 [file Table1.docx]

Supplementary Material

Rescue of follicle development after oocyte-induced ovary dysfunction and infertility in a model of premature ovarian infertility

Sairah Sheikh, Belinda KM Lo, Heidy Kaune, Jassimran Bansal, Anna Deleva and Suzannah A Williams

*** Correspondence:** Corresponding Author: suzannah.williams@wrh.ox.ac.uk

# Supplementary Figures and Tables

**Figure S1.** **Analysis of 3b primary follicles in Control and DM ovaries.** (A) Comparison of follicle, oocyte and granulosa cell area of primary 3b follicles between Control and DM at 8-days, 3-weeks, 6-weeks and 3-months. (B) Comparison of follicle and oocyte area of primary 3b follicles between Control and DM at 8-days, 3-weeks, 6-weeks and 3-months. Results are expressed as mean±SD. (C) Comparison of follicle, oocyte and GC area of primary 3b follicles within Control and DM ovaries at different ages. Results are expressed as mean±SEM. (D) The relationship between follicle, oocyte and GC area with GC number in primary 3b follicles in Control and DM was analysed at 8-days (open circles; Control n=3 mice, n=16 follicles, DM n=3 mice, n=34 follicles), 3-weeks (open squares; Control n=3 mice, n=21 follicles, DM n=3 mice, n=29 follicles), 6-weeks (open triangles; Control n=3 mice, n=17 follicles, DM n=3 mice, n=11 follicles) and 3-months (crosses; Control n=3 mice, n=28 follicles, DM n=3 mice, n=25 follicles). **P*≤0.05, ***P*≤0.01, ****P*≤0.001, *****P*≤0.0001.

**Table S1.** Statistical significance of analysis of relationship between follicle, oocyte and GC area with GC number in primary 3a follicles in Control and DM ovaries at 8-days, 3-weeks, 6-weeks and 3-months**.** Linear regression data for 3a follicles showing *P* values. If slopes are not significantly different, then differences between intercepts were calculated.

**Table S2.** Statistical significance of analysis of relationship between follicle, oocyte and GC area with GC number in primary 3b follicles in Control and DM ovaries at 8-days, 3-weeks, 6-weeks and 3-months**.** Linear regression data for 3b follicles showing *P* values. If slopes are not significantly different, then differences between intercepts were calculated**.**
